# Dimethyl Fumarate Protects Neural Stem/Progenitor Cells and Neurons from Oxidative Damage through Nrf2-ERK1/2 MAPK Pathway

**DOI:** 10.3390/ijms160613885

**Published:** 2015-06-17

**Authors:** Qin Wang, Sergei Chuikov, Sophina Taitano, Qi Wu, Arjun Rastogi, Samuel J. Tuck, Joseph M. Corey, Steven K. Lundy, Yang Mao-Draayer

**Affiliations:** 1 Department of Neurology, University of Michigan Medical School, 4015 Alfred Taubman Biomedical Sciences Research Building, 109 Zina Pitcher Place, Ann Arbor, MI 48109-2200, USA; E-Mails: qinwang@umich.edu (Q.W.); schuikov@umich.edu (S.C.); qiw@umich.edu (Q.W.); coreyj@umich.edu (J.M.C.); 2 Department of Internal Medicine, Division of Rheumatology, University of Michigan Medical School, Ann Arbor, MI 48109-2200, USA; E-Mails: sophinat@umich.edu (S.T.); sklundy@umich.edu (S.K.L.); 3 Geriatrics Research, Education, and Clinical Center (GRECC), VA Ann Arbor Healthcare Center, Ann Arbor, MI 48109-2200, USA; E-Mails: arastogi729@gmail.com (A.R.); samtuck@umich.edu (S.J.T.); 4 Department of Biomedical Engineering, University of Michigan, Ann Arbor, MI 48109-2200, USA; 5 Graduate Program in Immunology, Program in Biomedical Sciences, University of Michigan Medical School, Ann Arbor, MI 48109-2200, USA

**Keywords:** multiple sclerosis (MS), neural stem/progenitor cells (NPCs), dimethyl fumarate (DMF), MAPK, oxidative stress, neuroprotection

## Abstract

Multiple sclerosis (MS) is the most common multifocal inflammatory demyelinating disease of the central nervous system (CNS). Due to the progressive neurodegenerative nature of MS, developing treatments that exhibit direct neuroprotective effects are needed. Tecfidera™ (BG-12) is an oral formulation of the fumaric acid esters (FAE), containing the active metabolite dimethyl fumarate (DMF). Although BG-12 showed remarkable efficacy in lowering relapse rates in clinical trials, its mechanism of action in MS is not yet well understood. In this study, we reported the potential neuroprotective effects of dimethyl fumarate (DMF) on mouse and rat neural stem/progenitor cells (NPCs) and neurons. We found that DMF increased the frequency of the multipotent neurospheres and the survival of NPCs following oxidative stress with hydrogen peroxide (H_2_O_2_) treatment. In addition, utilizing the reactive oxygen species (ROS) assay, we showed that DMF reduced ROS production induced by H_2_O_2_. DMF also decreased oxidative stress-induced apoptosis. Using motor neuron survival assay, DMF significantly promoted survival of motor neurons under oxidative stress. We further analyzed the expression of oxidative stress-induced genes in the NPC cultures and showed that DMF increased the expression of transcription factor nuclear factor-erythroid 2-related factor 2 (*Nrf2*) at both levels of RNA and protein. Furthermore, we demonstrated the involvement of Nrf2-ERK1/2 MAPK pathway in DMF-mediated neuroprotection. Finally, we utilized SuperArray gene screen technology to identify additional anti-oxidative stress genes (*Gstp1*, *Sod2*, *Nqo1*, *Srxn1*, *Fth1*). Our data suggests that analysis of anti-oxidative stress mechanisms may yield further insights into new targets for treatment of multiple sclerosis (MS).

## 1. Introduction

Multiple sclerosis (MS) is a chronic neurological disease affecting a significant number of young adults [1,2]. Although the exact cause of MS is unknown, it is the most common multifocal inflammatory demyelinating disease of the central nervous system (CNS) [3,4,5]. The neurodegenerative process with damage to axons and oligodendrocytes is thought to be the cause of permanent neurological impairment and disability [6,7]. Due to the progressive neurodegenerative nature of MS, developing treatments that exhibit direct neuroprotective effects are needed. Currently, most available MS therapies are thought to exert their effects via immunomodulatory or immunosuppressive functions [5,8,9]. Although these treatments are effective at inhibiting immune cell-driven inflammation and reducing the relapse rate, they are ineffective at controlling the predominantly neurodegenerative processes that occur later in the disease course [10]. Methods of enhancing remyelination and decreasing axonal damage (*i.e.*, promoting neuroprotection) would be particularly useful for later stage and primary progressive MS forms.

Fumaric acid esters (FAE) have been used since 1959 as a treatment for psoriasis [11]. Tecfidera™ (BG-12) is an oral formulation of the FAE, containing the active metabolite dimethyl fumarate (DMF), which showed remarkable efficacy in lowering relapse rates in two phase III clinical trials of relapsing-remitting MS (RR-MS) treatment [12,13,14,15,16]. However, its mechanism of action is not yet well understood. It has been shown that FAE and its primary metabolite DMF and monomethyl fumarate (MMF) were able to activate the transcription factor nuclear factor-erythroid 2-related factor 2 (Nrf2) pathway and subsequently induce the expression of antioxidant proteins [17,18,19]. Oxidative stress plays a major role in multiple sclerosis (MS) and is readily apparent within experimental autoimmune encephalomyelitis (EAE), mouse model of MS, and MS lesions [15,20]. Invading leukocytes contribute to cell damage and demyelination by producing excessive amounts of cytotoxic mediators, including reactive oxygen species (ROS). Under normal conditions, ARE/EpRE (antioxidant/electrophile response element) is not active, but under stress Nrf2 activates ARE/EpRE [21]. Nrf2 is rapidly ubiquitinated and degraded by the help of the adaptor protein, kelch-like ECH-associated protein 1 (Keap1) [22]. Glutathione, a major intracellular ROS scavenger, is decreased within CNS inflammatory foci, and a variety of antioxidant proteins are increased in MS lesions [20]. Additionally, EAE in *Nrf2*^(−/−)^ mice is more severe and neuroprotective effects of DMF are absent in *Nrf2*^(−/−)^ mice [23]. Furthermore, it has been shown that administration of MMF could protect motor neurons and astrocytes against hydrogen peroxide (H_2_O_2_)-induced oxidative stress [23]. In a noninflammatory demylination model, DMF/MMF have little to no impact on demyelination or remyelination [24]. Other studies also have shown Nrf2-dependent cytoprotection of neurons and astrocytes, but did not demonstrate decreased infiltrates [25,26,27]. The therapeutic application of DMF in chronic EAE leading to a reduction in demyelination with relative preservation of myelin and axons suggests that the rescue of neurons and glial cells from oxidative stress-induced cell death is most likely through neuroprotection mediated by DMF-induced activation of Nrf2 pathway [23].

The observation of proliferating neural cells in the adult rat brain made the possibility of CNS regeneration more feasible. Neural stem/progenitor cells (NPCs) reside in the adult CNS and support neurogenesis and gliogenesis throughout life [8,28]. They are a heterogeneous population of perpetually self-renewing and multipotent cells that can spontaneously differentiate into neurons, astrocytes, or oligodendrocytes (post-mitotic daughter cells) [29]. The ability of NPCs to reconstitute neurons makes them an important target for therapy in MS, as various drugs may interact with stem cells to promote neural protection and/or remyelination. It has been thought that the benefits seen in the clinical symptoms of EAE mice after stem cell transplantation are mediated by neural stem cells and immune cell interactions that promote neuroprotection. NPCs transplantation holds significant promise as a novel treatment strategy for MS [30].

Neuroprotection has also been seen in excitotoxic neurodegeneration and Huntington’s disease. DMF has also been shown to improve lifespan, reduce behavioral deficits, and preserve striatal and motor cortex neurons in two different genetic models of Huntington’s disease in mice [31], suggesting broad neuroprotective properties. Damage of cortical neurons and motor neurons are the main cause of dysfunction and disability in MS patients. MS disease occurs early and progresses later in life. It is currently unknown whether DMF exerts its neuroprotective effects directly on differentiated neuronal cells and/or through activation of NPCs. Furthermore, the effect of DMF at the level of NPCs has not yet been reported. However, the mechanism of DMF action in these neurons, in terms of signaling pathways in MS, is not clearly understood.

In this study, we sought to investigate the neuroprotective effect of DMF in the context of two different neuronal cellular systems: NPCs and motor neurons. Our data showed that DMF protected NPCs in addition to differentiated motor neurons from oxidative damage (H_2_O_2_) through regulation of Nrf2, as well as several other novel genes involved in superoxide metabolism. Furthermore, the involvement of the signal transduction ERK1/2 MAPK pathway was identified in DMF-mediated neuroprotection.

## 2. Results

### 2.1. DMF Increased Frequency of Mouse NPCs in Vitro and Protected Rat NPCs and Motor Neurons from Oxidative Damage

**Figure 1:**
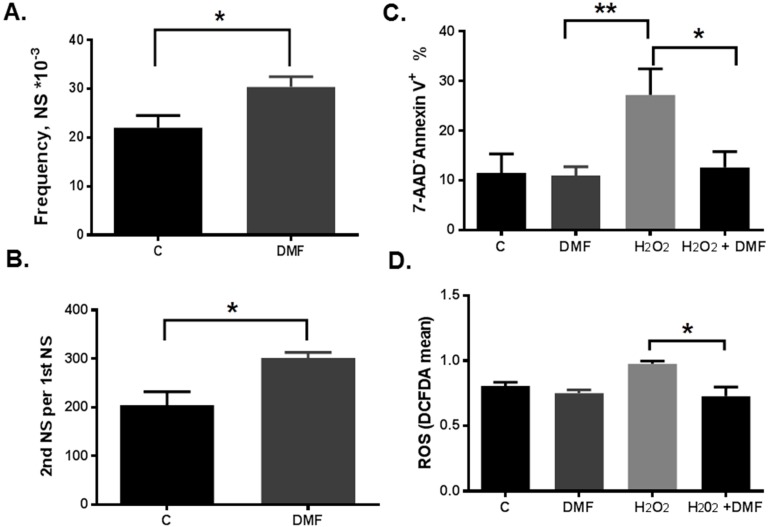
Dimethyl fumarate (DMF) increased self-renewal and reduced oxidative stress-induced apoptosis and ROS levels in neural stem/progenitor cells (NPCs). (**A**) Frequency of mouse neuronal stem/progenitor cells was calculated by dividing the number of neurospheres in the well by the number of plated cells with (DMF) or without (C) DMF treatment (Mean ± SEM were determined from seven replicate samples, * *p* = 0.027); (**B**) Self-renewal potential of mouse NPCs was calculated as number of secondary (2nd) neurospheres (NS) clonally derived from one primary (1st) neurosphere plated with (DMF) or without (C) DMF treatment (Mean ± SEM were determined from three replicate samples, * *p* = 0.03); (**C**) Rat NPCs were grown in adherent cultures in self-renewal medium. After treatment under indicated conditions with control (C), DMF alone (DMF) for overnight, then H_2_O_2_ alone (H_2_O_2_) and DMF together with H_2_O_2_ (H_2_O_2_ + DMF) for another 6 h, the NPC cells were trypsinized, then apoptosis assay was performed as described under “Material and methods”. Mean ± SEM were determined from three replicatesamples, * *p* = 0.015, ** *p* = 0.007); (**D**) Cells treated same as in “C” and then incubated with 5 µM 2′,7′-dichlorofluorescein diacetate (DCFDA) for 15 min at 37 °C and oxidative stress was measured as ROS binding to DCFDA and quantified by flow cytometry. Mean ± SEM were determined from three replicate samples, * *p* = 0.027.

To examine the neuroprotective effects of DMF, we first used mouse NPCs to determine whether DMF has any effects on neurosphere formation and self-renewal *in vitro*. In the neurosphere formation assay, we found that DMF increased the frequency of the multipotent neurospheres in culture with the frequency of 30 ± 2 comparing to 22 ± 2.6 in control per 1000 cells (Figure 1A). Next we tested whether DMF plays a role in regulation of stem/progenitor cells self-renewal. We calculated the number of secondary neurospheres clonally derived from one primary neurosphere plated under low cell density condition and found that DMF treatment increased the number of secondary neurospheres in the self-renewal assay with 302 ± 11 comparing to 204 ± 27 in control (Figure 1B).

To test whether DMF has neuroprotective effects with respect to oxidative damage, rat NPCs were under oxidative stress with hydrogen peroxide (H_2_O_2_) with or without DMF treatment, then an apoptosis assay was performed. DMF indeed decreased H_2_O_2_-induced apoptosis from 27.1% ± 3% to 12.6% ± 1.8%, effectively increasing the survival of rNPCs following oxidative stress treatment with H_2_O_2_. DMF treatment alone did not have any effect (11% ± 1%, Figure 1C). In addition, utilizing reactive oxygen species (ROS) assay, we showed that DMF reduced ROS production induced by H_2_O_2_ from 98 ± 0.002 to 0.73 ± 0.07 in rat NPCs (Figure 1D).

**Figure 2:**
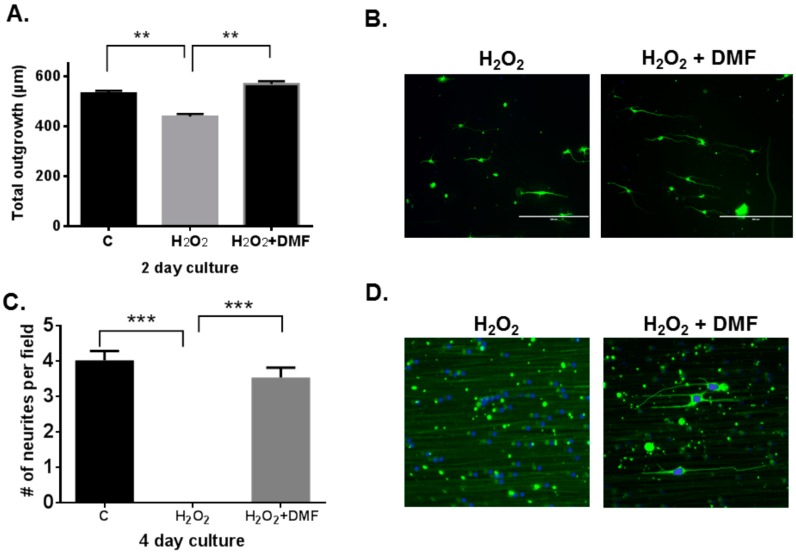
DMF promoted survival of rat motor neurons under oxidative stress. Isolated rat motor neurons were cultured on aligned, electrospun poly-l-lactic acid (PLLA) nanofiber scaffolds that direct neurite growth in straight lines along the fibers, allowing straightforward measurement of neurite length. Treatment with vehicle (control; labeled C), H_2_O_2_ alone (H_2_O_2_) and DMF together with H_2_O_2_ (H_2_O_2_ + DMF) was added after one day *in vitro*. Following fixation and staining, neurons were identified and their neurites were then counted and measured after two and four days *in vitro* (resulting in exposures of one and three days, respectively). Motor neuron survival was assessed by measuring the neurite length and counting the number of survived motor neurons on the slide view field. (**A**,**C**) mean ± SEM of three replicate measurements (*n* = 60–81); ** *p* < 0.01, *** *p* < 0.001 (**B**,**D**) representative images from Days 2 and 4 of culture. Scale bars = 200 µm.

To observe the efficacy of the neuroprotective features of DMF, dissociated rat motor neurons were cultured on electrospun poly-l-lactic acid (PLLA) nanofibers and the following features were measured: average number of neurites per image field, total neurite outgrowth, mean neurite length, and cell body area. The fibers serve as a substrate for growing the neurons since they direct neurite growth in a straight line, simplifying identification of neurites and neurite measurement. Neurons were exposed to oxidative stress with H_2_O_2_ after one day of culture and grown for either two or four days; half of these samples were treated with DMF. Both were compared to positive control samples that were unstressed and untreated. After two days of growth, total neurite outgrowth decreased on stress-treated neurons from 531 ± 16 to 438 ± 14 (Figure 2A), indicating poorer health of the neurons. However, addition of DMF abrogated this decrease and raised total neurite outgrowth from 438 ± 14 to 569 ± 16 (Figure 2A). No difference was observed amongst samples for cell body area or number of neurites per image field. This establishes that after one day of culture, neurons had begun to succumb to the stress of H_2_O_2_ treatment. After four days of growth (with three days of stress), no neurites were found on stressed motor neurons (Figure 2D). Addition of DMF protected neurons from stress as measured by average neurite number per image field (4 ± 0.3, *n* = 81, Figure 2C). This effect was also seen in all of our other measures: total neurite outgrowth, mean neurite length, and cell body area (data not shown). This substantiates the neuroprotective effects of DMF.

### 2.2. Mechanism of Neuroprotective Effects of DMF May Be through Regulation of the Anti-Oxidative Stress Gene Nrf2

**Figure 3:**
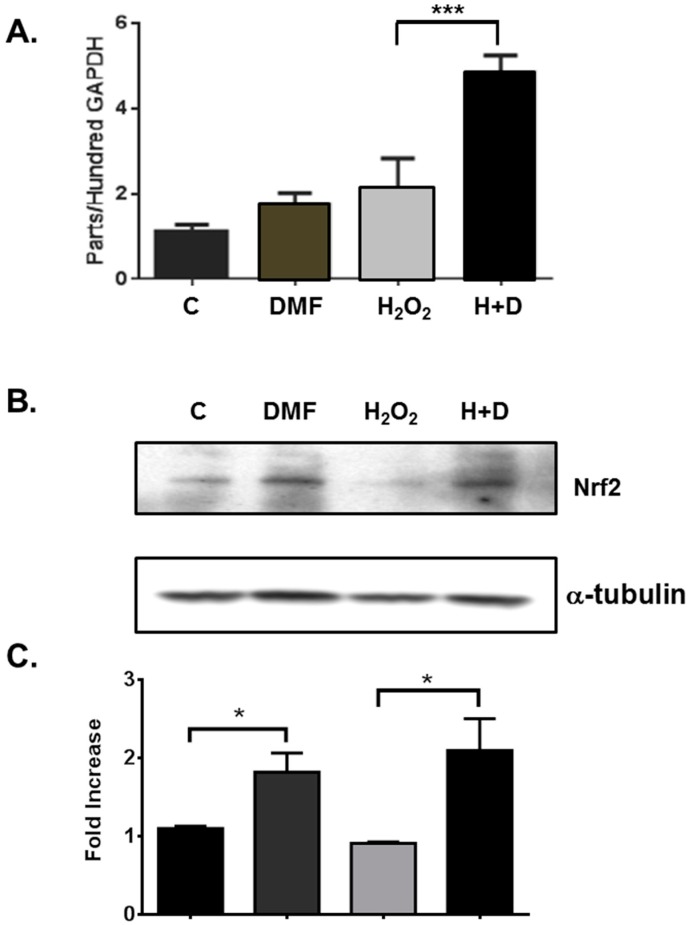
DMF increased Nrf2 RNA and protein expression in rat NPCs in the presence of oxidative stress. After treatment under indicated conditions with DMSO (C), DMF or H_2_O_2_ alone, or DMF and H_2_O_2_ together, Nrf2 RNA expression was determined using cDNAs prepared from rNPCs culture by RT-qPCR (**A**), *** *p* < 0.001; Nrf2 protein expression was assessed using whole cell lysates prepared from rNPCs cells by Western blot analysis (**B**) as described under “Material and Methods”; (**C**) Mean ± SEM were determined from 3–4 individual experiments. * *p* = 0.026.

The main mechanism of action that has been attributed to DMF involves direct effects on neuroprotection through activation of antioxidant response elements by the nuclear factor (erythroid-derived 2)-like 2 (Nrf2) pathway [23]. To determine whether *Nrf2* gene and protein were regulated by DMF in rNPCs, we used RT-qPCR and Western blot analysis to evaluate Nrf2 expression levels of DMF-treated rNPCs *in vitro*. Using cDNA or whole cell lysates prepared from rNPCs treated under different conditions with or without oxidative stress, total RNA and protein lysates were prepared for RT-qPCR and Western blot analysis for Nrf2 expression. We found that Nrf2 at both mRNA and protein levels was increased by DMF treatment and this effect was significantly enhanced in the presence of oxidative stress induced by H_2_O_2_ treatment (Figure 3). This suggests that the neuroprotective effects of DMF may be through regulation of the anti-oxidative stress gene *Nrf2*.

### 2.3. Mechanism of Involvement of ERK1/2 MAPK in DMF-Mediated Neuroprotection

**Figure 4:**
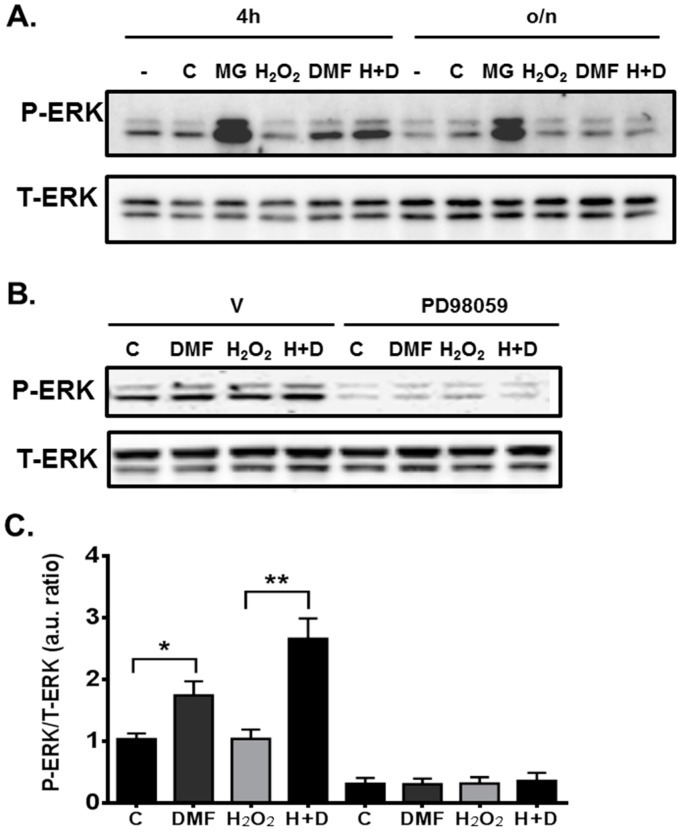
Involvement of ERK1/2 MAPK in DMF-mediated neuroprotection. (**A**) rNPCs cultures were serum starved in neurobasal medium with (24 h) or without (4 h) B-27 supplement and treated with medium alone (–), DMSO (C), MG132 (MG), DMF, H_2_O_2_, or both H_2_O_2_ and DMF (H + D). ERK1/2 phosphorylation was determined by Western blot analysis; (**B**) rNPCs cells were serum starved in neurobasal medium and treated with (PD98059) or without (V) the ERK1/2 inhibitor under the same treatment as “A” for 4 h; (**C**) ERK1/2 phosphorylation was presented as a ratio of normalized arbitrary units (a.u.) of phosphorylated ERK1/2 (P-ERK) over total ERK1/2 (T-ERK) in four individual experiments under same treatment as in “B”. * *p* = 0.028, ** *p* = 0.0042.

**Figure 5:**
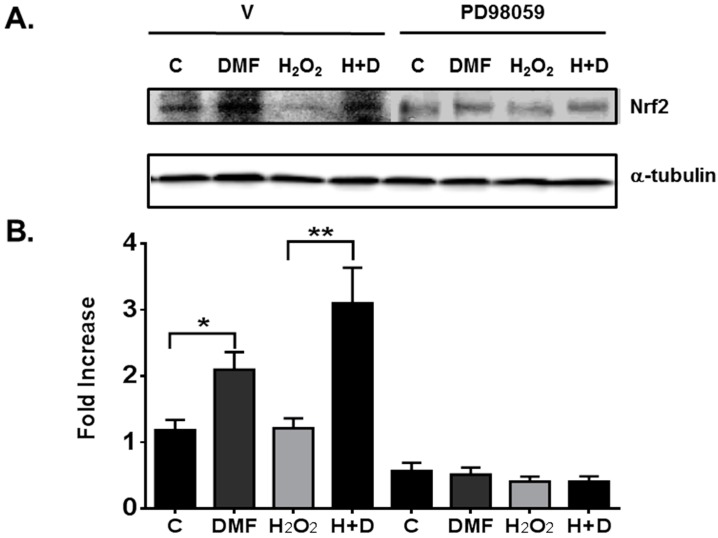
Effect of ERK1/2 inhibitor on Nrf2 protein level. (**A**) Whole cell lysates were prepared from the rNPCs cultures treated under various conditions with (PD98059) or without (V) the ERK1/2 inhibitor for overnight. Western blot analysis for Nrf2 protein expression was performed as described under “Material and Methods”; (**B**) Mean ± SEM were determined from 3–6 experiments. * *p* = 0.01, ** *p* = 0.0068.

The ERK1/2 MAPK pathway plays an important role in cell growth and survival [32]. To determine whether the ERK1/2 MAPK pathway is involved in DMF-mediated neuroprotection, we treated NPCs with H_2_O_2_ to induce oxidative stress. Due to the fact that Nrf2 is degraded through proteasomal cleavage, the proteasomal inhibitor, MG132, was used as a control [26,33]. MG132 dramatically increased ERK1/2 MAPK phosphorylation as expected (Figure 4A). DMF treatment alone increased phosphorylation of ERK1/2 MAPK from 1.0 ± 0.09 to 1.7 ± 0.23 and further induction was seen when treated with a combination of DMF and H_2_O_2_ for 4 h (2.7 ± 0.33). We also compared serum-starved NPCs that were treated for 4 h without B-27 supplement with NPCs treated overnight with B-27 supplement. Data showed that overnight treatment did not have an effect on ERK1/2 MAPK phosphorylation as seen with 4 h treatment except with the MG132 positive control. For the subsequent assay (Figure 4B,C), we performed only 4 h treatment. Since different kinases have been reported to regulate Nrf2 expression [16,34], to confirm the involvement of ERK1/2 MAPK pathway, we used MEK1 pathway (ERK1/2) MAPK inhibitor PD98059 [34], which completely blocked DMF- and H_2_O_2_-induced ERK1/2 phosphorylation including DMSO control (Figure 4B,C). The total ERK was not changed indicating that cell viability was not reduced. To further confirm the ERK1/2 MAPK involved in the Nrf2 pathway, the effect of the ERK1/2 inhibitor PD98059 on blocking Nrf2 protein level was investigated (Figure 5). PD98059 completely blocked DMF-induced Nrf2 protein expression. These data indicated that the mechanism of DMF-mediated neuroprotection may be through the involvement of ERK1/2 MAPK upstream of Nrf2 expression.

### 2.4. Identification of Additional Anti-Oxidative Stress Genes by SuperArray Gene Screen Technology

**Figure 6:**
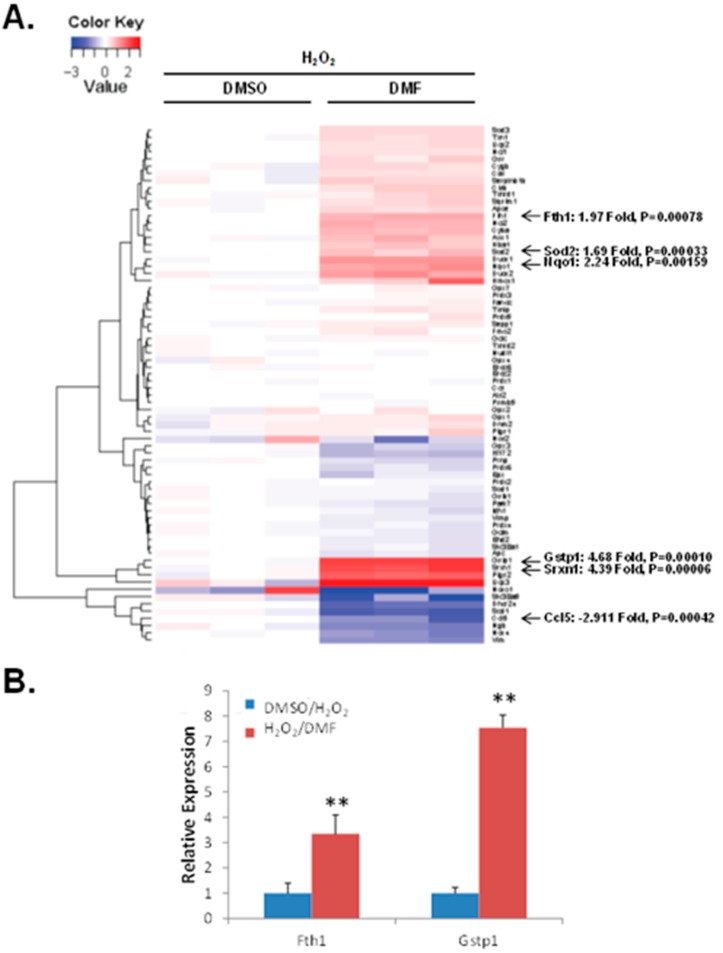
Identification of additional anti-oxidative stress genes by SuperArray gene screening technology. (**A**) SuperArray gene screen technology for anti-oxidative stress genes was performed as described under “Material and methods”. DMF significantly up-regulates gene expression of *Gstp1*, *Nqo1*, *Sod2*, *Srxn1*, and *Fth1*, with down-regulation of *Ccl5*; (**B**) Confirmation Real-Time qRT-PCR. Up-regulation of *Gstp1* and *Fth1* genes were further confirmed by Real-Time qRT-PCR. Mean ± SEM were determined from 3 replicate samples. ** *p* = 0.0015–0.0031.

When activated, Nrf2 protein dissociates from Keap1 complex, translocates into nucleus, and then activates ARE-mediated phase II antioxidant gene expression [35]. To further determine whether DMF has an effect on up- or down-regulation of antioxidant gene(s) expression, we utilized Oxidative Stress PCR SuperArray gene screen technology to identify additional anti-oxidative stress genes under our conditions with the treatment of DMF and oxidative stress. Our results showed that under oxidative stress with H_2_O_2_, DMF significantly up-regulated gene expression of genes including *Gstp1* (Glutathione *S*-transferase pi 1-, 5-fold increase), *Nqo1* [NAD(P)H dehydrogenase, quinone 1-, 2-fold increase], *Sod2* (Superoxide dismutase 2, mitochondrial, 2-fold increase), *Srxn1* (Sulfiredoxin1 homolog, 4-fold increase), and *Fth1* (Ferritin, 2-fold increase) (Figure 6A), which all play roles in reducing oxidative stress by breaking down ROS. Intriguingly, in NPC cultures, DMF also showed a trend of down-regulating genes including *Ccl5* [Chemokine (C–C motif) ligand 5-, 3-fold decrease], which is a ligand of CCR1, CCR3, and CCR5 with the potential to reduce the recruitment of leukocytes into inflammatory sites (Figure 6A). Using Real-Time qRT-PCR analysis, *Fth1* and *Gstp1* gene up-regulation were further confirmed (Figure 6B).

## 3. Discussion

Our current study has shown that DMF protects different neuronal cell types including neural stem/progenitor cells (NPCs) and differentiated neurons (motor neurons) from oxidative damage through regulation of Nrf2 involving ERK1/2 MAPK pathway. Key *Nrf2* target genes and genes involved in superoxide metabolism were demonstrated to be induced by DMF in rNPCs. The possible role of this mechanism in reducing oxidative stress by breaking down ROS suggests that analysis of anti-oxidative stress mechanisms in NPCs may yield further insights into new targets for treatment of MS.

Inflammation and oxidative stress are thought to promote CNS tissue damage in multiple sclerosis by affecting demyelination. Recent studies had described a potential neuroprotective mechanism of action for the fumaric acid ester, dimethyl fumarate (DMF), and its primary metabolite, monomethyl fumarate (MMF), on neurons, astrocytes and glial cells demonstrated by *in vitro* and *in vivo* data [23,31]. Protection of oligodendrocyte and neurons from irreversible damage in CNS may exert neuroprotective effects both directly and indirectly in the periphery and CNS. It has been shown that DMF and MMF are able to activate Nrf2, the key transcription factor regulating the antioxidant response and induce expression of antioxidant proteins [19]. Furthermore, administration of MMF could protect motor neurons and astrocytes against H_2_O_2_-induced oxidative stress [23]. These results may point to an underlying functional cellular substrate for the neuroprotective response observed in mouse EAE, in which DMF treatment reduced oxidative damage and consequential nerve fiber demyelination, resulting in greater axonal preservation and improved motor function [23,36]. Different CNS cell types were used to demonstrate the DMF- and MMF-dependent stabilization of Nrf2, indicating the relevance of these compounds in treating diseases that result in the degeneration of CNS cells. Reports have shown that DMF exerted anti-inflammatory and prometabolic effects in a variety of cell types in primary astrocytes and C6 glioma cells by modifying glutathione (GSH) levels that can induce expression of the anti-inflammatory protein HO-1 (heme oxygenase-1) [19]. DMF treatment increased nuclear levels of Nrf2 in astrocytes but not C6 cells. DMF suppressed inflammatory activation in astrocytes and C6 glioma cells, but with distinct mechanisms, different dependence on GSH, and different effects on transcription factor activation. The effect of DMF on NPCs was not previously demonstrated. We add to this data that DMF directly enhanced NPCs neurosphere growth, and decreased oxidative stress-induced apoptosis in both NPCs and neurons treated with H_2_O_2_.

It has been suggested that NPCs and perhaps neurons are capable of responding to DMF treatment, leading to induction of a prototypical Nrf2-dependent antioxidant response. One report also showed that DMF promoted cytoprotection of central nervous system cells against oxidative stress via Nrf2 [26]. Some other studies have shown that antioxidant response proteins like Nrf2 are elevated in MS lesions [20,37]. However, the degree of intrinsic antioxidant induction is apparently insufficient to ultimately prevent disease progression. Neural stem/progenitor cells (NPCs) proliferate and produce new neurons in neurogenic areas throughout the lifetime. While these cells represent potential therapeutic treatment of neurodegenerative diseases, regulation of neurogenesis is not completely understood [38]. Our observations have confirmed that DMF can activate the Nrf2 pathway in rat NPCs and stabilize levels of Nrf2 protein, resulting in the accumulation of active transcription factor, thereby conferring neuroprotective responses against oxidative stress.

All cells have an intrinsic mechanism for combating reactive oxygen species (ROS) that is dynamically controlled through the actions of Nrf2, a transcription factor that is the principal regulator of the phase II cellular antioxidant response [39]. The studies described here demonstrated that DMF treatment can decrease apoptosis and increase cell survival in motor neurons under H_2_O_2_ oxidative stress which further supported the DMF neuroprotective effects on primary cultures of motor neurons and suggests a potential role for cell type-specific neuroprotective responses.

In addition to characterization of Nrf2 pathway activation in NPC cells, the potential upstream and downstream functional mechanism by which DMF may promote resistance to oxidative stress has not been identified. Though the involvement of mitogen-activated protein kinases (MAPKs) in the Nrf2 pathway has been reported, different subgroups of MAPKs are involved, and their exact functions differ depending on cell types [35,40]. MAPKs play crucial roles in cellular processes, including proliferation, stress responses, apoptosis, and immune defense [41]. Some reports have shown that Nrf2 protein accumulation has been induced by activation of MAPKs. ERK1/2 is one of three major subgroups of MAPKs. An ERK pathway inhibitor prevented Nrf2 phosphorylation at Ser40 to retard Nrf2 nuclear translocation, thus decreasing antioxidant gene transcription [35,40,42]. There is no previous report on DMF neuroprotection via ERK1/2 MAPK-induced Nrf2 accumulation in neurons and NPCs. Here we showed that DMF increased Nrf2 accumulation and oxidative stress enhanced this effect through ERK1/2 phosphorylation which was completely abolished upon treatment with the ERK1/2 inhibitor, PD98059 [43,44]. The neuroprotective action of DMF on NPCs is therefore likely through increasing Nrf2 accumulation by activation of the ERK1/2 signaling pathway. This does not exclude the possibility that other signaling pathways may also be involved, such as the NFκB pathway, which also decreases apoptosis of neurons, astrocytes, microglia and oligodendrocytes [19,45,46]. Currently, we cannot rule out the possibility that DMF may act as a Keap1 inhibitor that leads to the accumulation of Nrf2 protein by decreasing proteasomal degradation [47,48,49,50]. This neuroprotective role of DMF may explain the decreased rate of brain atrophy and the slowed rate of disease progression in patients with MS treated with Tecfidera™ [51,52]. Elucidation of this pathway suggests a potential role for cell type-specific neuroprotective responses.

Multiple genes have been reported to be candidates for direct targets of Nrf2 activation in different cell types and tissues which are enhanced only in the presence of the *Nrf2* gene [53,54]. The roles of these *Nrf2* target genes in protection from oxidative stress were unknown for NPCs. To our knowledge, this is the first report to screen *Nrf2* target genes and genes involved in superoxide metabolism in rat NPCs. Hence this is the first report in which five *Nrf2* targeted genes (*Fth1*, *Gstp1*, *Sod2*, *Nqo1* and *Srxn1*) have been identified as strong candidates for DMF-mediated Nrf2 activation in rat NPCs. Under oxidative stress, DMF significantly up-regulated the expression of these genes, which all play roles in reducing oxidative stress by breaking down ROS.

Sod2 is a critical component of the mitochondrial pathway for detoxification of O_2_^−^ and targeted disruption of this locus leads to embryonic or neonatal lethality in mice [55]. Gstp1 is a phase II detoxification enzyme that conjugates both endogenous and exogenous compounds to glutathione to reduce cellular oxidative stress. Decreased expression of Gstp1 has recently been implicated in dopamine neuron degeneration in Parkinson’s disease progression [56]. Nqo1 has been shown to be upregulated in Sulforaphane protection of cortical neurons against 5-*S*-cysteinyl-dopamine-induced toxicity through the activation of the ERK1/2-Nrf2 pathway [57]. Srxn1 is an endogenous antioxidant protein that has neuroprotective effects. While the mechanisms of Srxn1 in oxidative stress have not been well studied, it has been shown that Srxn1 protects PC12 cells against oxidative stress induced by hydrogen peroxide [58]. Srxn1 expression has also been shown to mediate protective effects of brain-derived neurotrophic factor (BDNF) against neurotoxicity in primary rat cortical cultures induced by 3-nitropropionic acid (3-NP), a mitochondrial complex II inhibitor [59]. *Fth1* gene has been implicated in protection against neuronal cell death under various neurodegenerative stresses, such as hypoxia-ischemia and oxidative stress and its increased expression may have protective effects against neural damage that could otherwise result in cell death [60]. DMF also showed a trend of down-regulation of Ccl5, a ligand of CCR1, CCR3, and CCR5, which could potentially reduce the recruitment of leukocytes into CNS inflammatory sites, thereby decreasing cell trafficking and inflammation [61]. In particular, abnormal Ccl5 expression was detected in the cerebrospinal fluid (CSF) of patients suffering from MS and in the CNS of EAE mice [62,63,64,65,66] implicating this chemokine in the onset of the demyelinating disease(s).

## 4. Materials and Methods

All experiments were done in accordance with the NIH Guide for Care and Use of Laboratory Animals, as approved by the R&D Service of the VA Ann Arbor Healthcare Center on 21 November 2012 (ACORP 2012-090604) and the University Committee on Use and Care of Animals (UCUCA) of the University of Michigan on 6 June 2013 (PRO 00004815).

### 4.1. Preparation of Rat NPCs and Mouse Neurospheres

NPCs were isolated based on neurosphere formation and confirmed through staining with the NPC marker nestin as previously described [67,68,69]. Rat embryonic E15-18 pups were decapitated, and the heads were placed into a petri dish with ice-cold HBSS (Ca^2+^, Mg^2+^ free). The whole brain without cerebellum was dissected and washed with fresh HBSS solution. It was then digested with 25 µg/mL DNase I in ice-cold HBSS and filtered through a cell strainer (70 µm, Falcon, BD Biosciences, San Jose, CA, USA). Cells were counted and plated onto 100 mm dishes pre-equilibrated with self-renewal media (SRM) containing DMEM, Neurobasal-A media, 2-mercaptoethanol, Chick Embryo Extract (CEE), 1× N-2 supplement, 2× B-27 supplement, FGF (20 ng/mL), EGF (20 ng/mL), and Penicillin-Streptomycin. Mouse neurosphere preparation and growth were carried out as described previously [69]. Briefly, neonatal mouse brain was dissected and washed with fresh cold HBSS solution. Two coronal slices (as thin as possible, ~1–2 mm) were cut starting at the olfactory bulb. The lateral wall of the lateral ventricle was removed and mechanically triturated in a 15 mL conical tube with 1:3 dilution of trypsin. These cells were incubated for 4 min at 37 °C, quenched in two volumes of medium containing 25 µg/mL DNase I and spun at 210× *g* at 4 °C for 5 min. The pellets were re-suspended in ice-cold HBSS, counted, and plated at a density of 1000 cells/well onto 6-well low binding tissue culture plates pre-equilibrated with SRM. After 8–10 days in culture the neurospheres were counted, measured, and selected for the self-renewal assay. The frequency of neurospheres was calculated with the numbers of neurospheres formation from originally plated 1000 cells. To assay self-renewal potential, individual primary neurospheres (>50 μm in diameter) were dissociated and re-plated at clonal density in non-adherent secondary cultures.

### 4.2. Primary Rat Motor Neurons Preparation and Culture

Primary motor neurons were cultured as previously described [70]. Briefly, perineural membranes were removed from spinal cords of E15 Sprague-Dawley pregnant rats and the tissue was chopped into 2-mm pieces. Cells were dissociated by incubating in 0.25% trypsin/EDTA for 15 min at 37 °C followed by gentle trituration for one minute with a serum-coated, fire-polished glass pasteur pipette. Motor neurons were isolated over 9% Optiprep in L-15 media by centrifugation at 1000× *g* for 15 min and then collected by taking 2 mL of supernatant. Cells were washed in L-15 media, then resuspended and plated in culture medium. Defined culture media consisted of Neurobasal (Invitrogen, Carlsbad, CA, USA) supplemented with 2× B27 and the following additives: 2.5 mg/mL albumin, 2.5 µg/mL catalase, 2.5 µg/mL superoxide dismutase, 0.01 mg/mL transferrin, 15 µg/mL galactose, 6.3 ng/mL progesterone, 16 µg/mL putrescine, 4 ng/mL selenium, 3 ng/mL b-estradiol, 4 ng/mL hydrocortisone, and 1× penicillin/streptomycin/neomycin. l-Glutamine (2 µM) was added to the medium immediately before plating. Cells were counted and plated at a density of 25 cells/mm^2^, so that neurons would not contact one another.

### 4.3. Immunocytochemistry and Morphological Analysis

Immunohistochemistry techniques were performed as previously described [70]. Briefly, cells were fixed in 4% paraformaldehyde at room temperature for 30 min. To block nonspecific antibody binding, samples were incubated in 2% goat serum/2.5% BSA/0.05% Triton-X-100 in 1× PBS for 30 min. Primary antibody (anti-β-Tubulin III diluted 1:1000, Sigma, St. Louis, MO, USA) was prepared in 10% goat serum/2.5% BSA/0.05% Triton-X-100/0.1% sodium azide in 1× PBS and incubated with cells overnight. Cells were then washed in 1× PBS and incubated in the secondary antibody Goat anti-mouse IgG (FITC) diluted 1:200 (Sigma) for 4 h. Prolong Gold (Molecular Probes/Invitrogen, Carlsbad, CA, USA), an antifade agent with 4′,6-diamidino-2-phenylindole (DAPI), was used to stain nuclei. The neurons were imaged using an EVOS fluorescent microscope and analyzed with Metamorph imaging software (Molecular Devices, LLC. Sunnyvale, CA, USA). Neurite number, total neurite growth, mean neurite growth, and cell body area were all recorded.

### 4.4. Treatments

Dimethyl fumarate (DMF) and Hydrogen peroxide solution (H_2_O_2_, 30% *w*/*w* in H_2_O, contains stabilizer) was purchased from Sigma–Aldrich (St. Louis, MO, USA). DMF was dissolved in DMSO and aliquots were kept in dark in −20 °C freezer. H_2_O_2_ was kept at 4 °C refrigerator. Newly opened bottle of H_2_O_2_ was used and freshly prepared and diluted H_2_O_2_ stock solution was immediately added into cell culture medium to achieve the desired working concentration. The concentration responses for both H_2_O_2_ (ranging from 0, 10, 20, 40, 80, and 100 μM) and DMF (ranging from 0, 10, 20, and 40 μM) and time course for both H_2_O_2_ and DMF (ranging from 4, 6, 18, 24, 48, and 72 h) were studied in the culture medium and then the optimal conditions (40 µm H_2_O_2_ and 10 µm DMF) for studying DMF neuroprotective effect under H_2_O_2_-induced oxidative stress were determined for subsequent experiments. In most experiments unless otherwise noted, the cultures were divided into four different groups which were treated in the following way: (1) Control with same concentration of DMSO used to dissolve DMF; (2) DMF alone; (3) H_2_O_2_ alone with same concentration of DMSO; (4) DMF + H_2_O_2_. At least three separate cultures were set up for each experiment.

### 4.5. Survival Assay

Motor neurons were isolated from E15 pregnant Sprague Dawley rats and plated onto poly (lactic-*co*-glycolic acid) (PLGA) solvent-casted, poly-l-lactic acid (PLLA) electrospun nanofibers coated with poly-l-lysine (PLL) [70]. Three separate groups of cells were cultured: a control group of motor neurons on fibers treated with 0.1% DMSO, a group of cells cultured with 40 µM H_2_O_2_ (the stress group), and a group of cells cultured with both 40 µM H_2_O_2_ and 20 µM DMF (the treatment for this experiment). Cells were cultured for one day before any addition of treatment (DMF) or stress H_2_O_2_. In a separate experiment, cells were subjected to 20 µM DMF alone and did not show any difference in growth when compared to controls. Concentrations of H_2_O_2_ and DMF were determined in pilot experiments on neurons for optimal destructive/neuroprotective results.

### 4.6. Electrospinning

The electrospinning methods were similar to those previously described [70]. Briefly, 0.4 g of poly-l-lactic acid (PLLA) was dissolved in a total volume of 10 mL in parts 9:1 chloroform: dimethylformamide, to yield a 4.4% *w*/*v* solution. The mixture was loaded into a 3 mL syringe with a 22 gauge blunt-tip needle and placed on a syringe pump set to 0.22 mL/h. The tip of the needle was protruded through the center of a 10 × 10 cm folded aluminum sheet. The rotating disc collector was placed 30 cm away, such that the axis of rotation was perpendicular to the syringe. A high voltage power supply applied a 20 kV charge to the aluminum sheet via an alligator clip. A second power supply applied a counter charge of −2 kV to the rotating disc via a wire brush. Fibers were collected for 5 min or until a desired density was obtained.

### 4.7. Apoptosis Assay

Rat NPCs were grown in adherent cultures in self-renewal medium. Cultures were pre-treated with indicated concentration of DMF (or DMSO control) overnight. After H_2_O_2_ treatment, the NPC cells were trypsinized, washed with 1× Annexin binding buffer and stained with APC-Annexin V kit (BD Biosciences, San Jose, CA, USA), according to the manufacturer’s instructions. 7-AAD viability staining solution (BioLegend, San Diego, CA, USA) was used to determine the necrotic cells. Staining was analyzed by flow cytometry and the apoptotic cell population was calculated by the percent of Annexin V positive cells.

### 4.8. ROS Assay

Rat NPCs were grown in adherent cultures overnight following pretreatment with DMF or DMSO (control) in minimal medium containing Neurobasal-A medium plus 1:1:2 ratio of penicillin/streptomycin:l-Glutamine:glucose at 1:100 dilution. After induction of oxidative stress by addition of H_2_O_2_, cells were incubated with 5 µM 2′-7′-dichlorofluorescein diacetate (CM-H2DCFDA, Life Technology, Grand Island, NY, USA) for 15 min at 37 °C, washed, and the ROS levels were measured by flow cytometry [71].

### 4.9. Western Blot Analysis for Nrf2 Protein

After treatment, whole cell lysates were prepared in RIPA lysis buffer containing protease inhibitors, subjected to SDS-PAGE on an 8% mini-gel and transferred to Immobilon™-P transfer membrane as previously described [33]. The membrane was blocked and cut at 75 kDa marker. The upper part of membrane was incubated with 1 µg/mL anti-Nrf2 antibody (R&D Systems Tools for Cell Biology Research) [72] and the bottom part of membrane was incubated with anti-α tubulin (Sigma) as a loading control at a 1:10,000 dilution for overnight in a 4 °C cold room. After three consecutive washes with TBS plus 0.1% Tween 20 (10 min for each), the membranes were incubated with 1:10,000 secondary antibody (anti-mouse IgG-HRP, Sigma) for 1 h at room temperature. Prestained SDS-PAGE protein standards (Bio-Rad, Hercules, CA, USA) were used to determine the size of detected proteins. Proteins were visualized by chemiluminescence with SuperSignal West Pico (Pierce, Grand Island, NY, USA) and exposed to X-ray film.

### 4.10. ERK1/2 Phosphorylation Assay

Rat NPCs were plated in a 24-well plate and changed to serum-free medium for 4 h before the assay. The ERK1/2 phosphorylation assay was performed as previously described [43]. Briefly, cells were washed and removed from the wells, boiled, and then subjected to electrophoresis using a 12% SDS-PAGE mini-gel, followed by transfer to an Immobilon™-FL membrane (Fisher, Loughborough, UK) for Western blotting. After blocking, the blot was probed with mouse anti-phospho-p44/42MAPK (Thr202/Tyr204) antibody and rabbit anti-p44/42 MAPK antibody (Cell Signaling Technology, Danvers, MA, USA) overnight on a rocking shaker in a 4 °C cold room. Then, the blot was incubated with the secondary antibodies, anti-mouse IRdye 680 RD and anti-rabbit IRdye 800 CW, for 1 h at room temperature. Finally, images were acquired using an Odyssey FC imaging system (Li-COR Biosciences, Lincoln, NE, USA) and quantified using the manufacturer’s analysis program. The ERK1/2 phosphorylation was calculated as the ratio of normalized arbitrary units (a.u.) of phosphorylated ERK1/2 over total ERK1/2.

### 4.11. RNA Purification and Gene Expression Analysis

Rat NPCs were grown in adherent cultures and treated under various conditions described above. Then the total RNAs were prepared using RNeasy RNA purification kit (Qiagen, Valencia, CA, USA) according to the manufacturer’s instruction. RNA integrity was assessed with the Agilent 2100 Bioanalyze at the University of Michigan microarray facility. RNA concentration was quantified using Nanodrop (Thermo Scientific, Grand Island, NY, USA) and the samples were tested for absence of genomic DNA contamination. Gene expression of oxidative stress related genes were assessed using Oxidative Stress PCR SuperArray [73]. RNA samples (80 ng each) were reverse-transcribed to cDNA using the SuperArray RT^2^ First Strand Kit (Qiagen, Valencia, CA, USA). cDNA samples were next subjected to Oxidative Stress PCR SuperArray. PCR amplifications (Applied Biosystems 7900HT, Thermo Scientific, Grand Island, NY, USA) were completed with PCR plates (Superarray Cat No. PAHS-031A) assaying for 84 neurotrophic factors and receptors and five houkeeping genes. Results were quantified using (2^−ΔΔ*C*t^) method and expressed as a fold difference up/down with respect to the control condition.

### 4.12. Real-Time RT-qPCR

The candidate genes that were either up- or down-regulated by DMF treatment were confirmed by real time-quantitative polymerase chain reaction (RT-qPCR) using Applied Biosystems 7500 (Thermo Scientific, Grand Island, NY, USA). Amplification consisted of 40 cycles (95 °C for 15 s and 60 °C for 1 min) with approximately 15 ng/μL cDNA, *TaqMan* Master Mix and specific primer pairs were purchased from Applied Biosystems (assay on demands primers).

### 4.13. Statistical Analysis

Experimental data was analyzed using GraphPad Prism version 6.0 (GraphPad Software Inc., San Diego, CA, USA). The unpaired *t*-tests were performed for all experiments and values were expressed as mean ± SEM from at least three individual experiments. *p*-Values were considered significant at * *p* < 0.05 and highly significant at ** *p* < 0.01 or *** *p* < 0.001.

**Figure 7:**
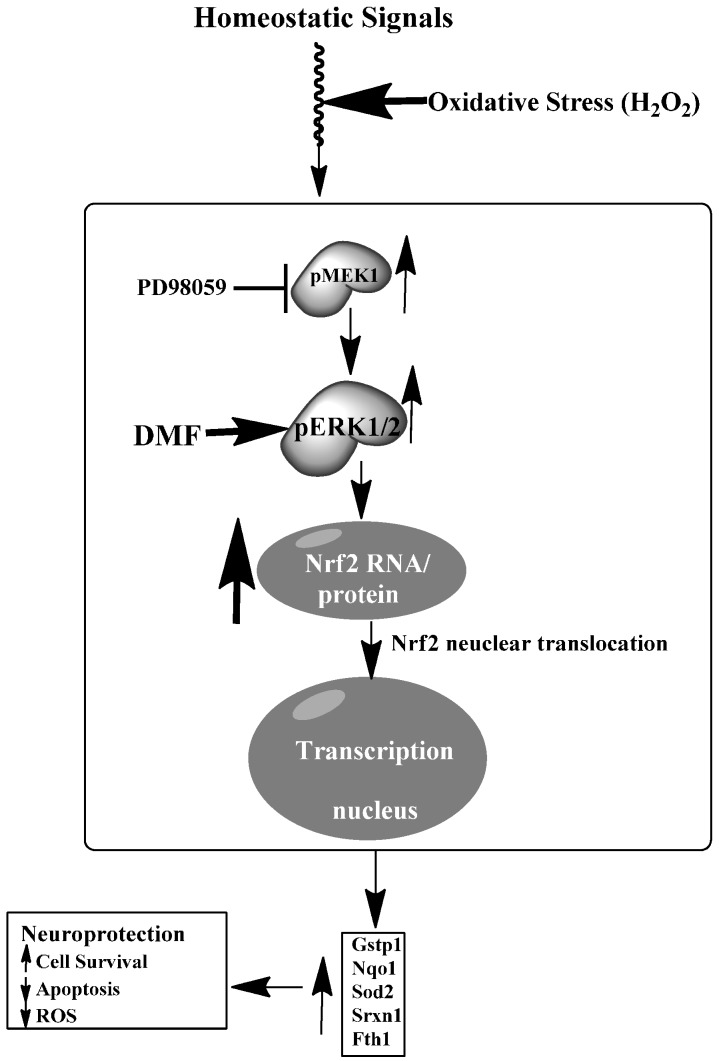
Schematic model of DMF-induced neuroprotection in rat NPCs and differentiated neurons. Under oxidative stress (H_2_O_2_), DMF enhances Nrf2-mediated antioxidant response gene transcription through activation of ERK1/2 MAPK signaling pathway leading to neuroprotection in rNPCs and differentiated neurons.

## 5. Conclusions

DMF protected neural stem/progenitor cells and differentiated neurons from oxidative damage through regulation of Nrf2 involving the ERK1/2 MAPK pathway. We propose that under oxidative stress (H_2_O_2_), DMF enhances Nrf2-mediated antioxidant response gene transcription through activation of the ERK1/2 signaling pathway leading to neuroprotection in rat NPCs and differentiated neurons (Figure 7). The key *Nrf2* target genes and genes involved in superoxide metabolism are involved in reducing oxidative stress by breaking down ROS, decreasing apoptosis and increasing survival. Investigating the anti-oxidative stress mechanisms may yield further insights into new targets for treatment of multiple sclerosis (MS).

## Abbreviations

MS: multiple sclerosis
MAPK: mitogen-activated protein kinase
ERK1/2: extracellular-signal-regulated kinases 1/2
FGF: recombinant human basic fibroblast growth factor 146 aa
EGF: epidermal growth factor
RIPA: radio-immunoprecipitation assay
rNPCs: rat neural stem/progenitor cells
ROS: reactive oxygen pecies
CNS: central nervous system
FAE: Fumaric acid esters
MMF: monomethyl fumarate
DMF: dimethyl fumarate
H_2_O_2_: hydrogen peroxide
Nrf2: transcription factor nuclear factor-erythroid 2-related factor 2
EAE: experimental autoimmune encephalomyelitis
MOG: myelin oligodendrocyte glycoprotein
ARE/EpRE: antioxidant/electrophile response element
IL-10: interlukin-10
Keap1: kelch-like ECH-associated protein 1
CEE: chick embryo extract
PLGA: poly lactic-*co*-glycolic acid
PLLA: poly-l-lactic acid
PLL: poly-l-lysine
SRM: self-renewal medium
EGFP: enhanced green fluorescent protein
CM-H2DCFDA: chloromethyl derivative of 2′,7′-dichlorofluorescein diacetate
RT-qPCR: real time quantitative reverse transcription polymerase chain reaction
GSH: glutathione
HO-1: heme oxygenase-1
DMSO: dimethyl sulfoxide
FITC: fluorescein isothiocyanate
DAPI: 4′,6-diamidino-2-phenylindole
*Gstp1*: glutathione *S*-transferase pi 1
*Nqo1*: NAD(P)H dehydrogenase, quinone 1
*Sod2*: superoxide dismutase 2
*Srxn1*: sulfiredoxin 1 homolog
*Fth1*: ferritin 1
*Ccl5*: chemokine (C–C motif) ligand 5
